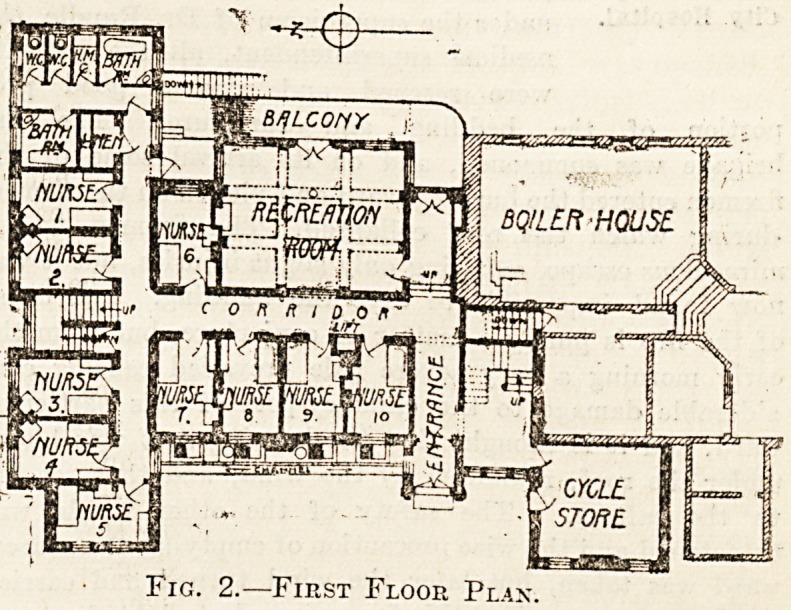# Nurses' Home, Salop Infirmary, Shrewsbury

**Published:** 1911-04-29

**Authors:** 


### Hospital Architecture and Construction.

[Communications on this subject should be marked "Architecture" in the left-hand top corner of the envelope.]

### NURSES' HOME, SALOP INFIRMARY, SHREWSBURY.

The space at the disposal of the architect for planning this building was very restricted. With the laundry and boiler-house on one side and adjoining premises closely contiguous on the other, his task was not an easy one; but the plan shows that the difficulties have been successfully overcome.

To get the required number of rooms it was necessary to carry the building up five storeys, and the only point for regret is that no lift should have been provided. Possibly expense was the deciding factor, but with a building of this height we are justified in saying that it would have been a humane provision.

Accommodation is provided for thirty-five sisters and nurses and twenty-five servants. There are two large siting-rooms and a large recreation-room in the basement. The rooms are well-proportioned and lighted, and each room is provided with a fireplace. There are two staircases, and communication is provided with the Infirmary by a corridor at the first level.

The architect is Mr. A. E. Lloyd Oswell, of Shrewsbury.

Fig. 1.—Ground Floor Plan.

Fig. 2.—First Floor Plan.

## Figures and Tables

**Fig. 1. f1:**
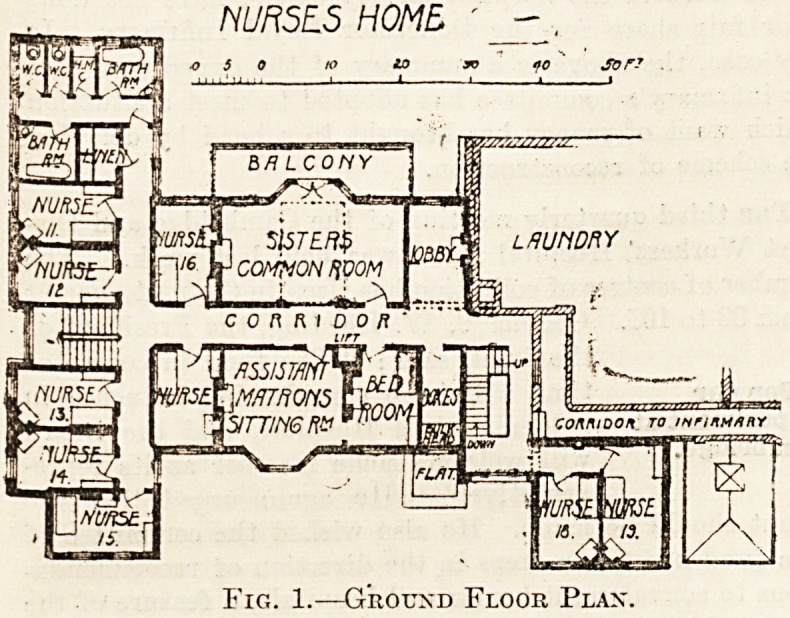


**Fig. 2. f2:**